# Booster COVID-19 Vaccinations Among Persons Aged ≥5 Years and Second Booster COVID-19 Vaccinations Among Persons Aged ≥50 Years — United States, August 13, 2021–August 5, 2022

**DOI:** 10.15585/mmwr.mm7135a4

**Published:** 2022-09-02

**Authors:** Hannah E. Fast, Bhavini Patel Murthy, Elizabeth Zell, Lu Meng, Neil Murthy, Ryan Saelee, Peng-jun Lu, Yoonjae Kang, Lauren Shaw, Lynn Gibbs-Scharf, LaTreace Harris

**Affiliations:** ^1^Immunization Services Division, National Center for Immunization and Respiratory Diseases, CDC; ^2^Stat-Epi Associates, Inc., Ponte Vedra Beach, Florida; ^3^CDC COVID-19 Emergency Response Team; ^4^General Dynamics Information Technology Inc., Falls Church, Virginia.

COVID-19 vaccine booster doses provide enhanced protection against SARS-CoV-2 infection, emergency department visits, hospitalization, and death (*1*–*3*). As of May 19, 2022, all fully vaccinated persons aged ≥5 years are recommended to receive a booster dose when eligible; selected populations, as determined by age and immunocompromise status, are also eligible for a second booster or an additional dose to complete a primary COVID-19 vaccination series (*4*). Data on COVID-19 vaccine doses administered during August 13, 2021**–**August 5, 2022, and submitted to CDC from 50 states and the District of Columbia (DC) were analyzed to assess booster and second booster vaccination coverage among eligible populations, by age group, sex, race and ethnicity, urban-rural classification, and the primary series vaccine product received. For this analysis, primary series completion was defined as receipt of 2 mRNA (i.e., mRNA-1273 [Moderna] or BNT162b2 [Pfizer-BioNTech]) COVID-19 vaccine doses or 1 Ad26.COV.S (Janssen [Johnson & Johnson]) COVID-19 vaccine dose because data were not available to identify immunocompromised persons who might have received an additional primary dose. Among 214.4 million eligible persons aged ≥5 years, 106.3 million (49.6%) received a booster dose, and booster dose coverage increased with age. Booster dose coverage was lowest for children, adolescents, and adults aged 18–39 years; males; non-Hispanic Black or African American (Black), Hispanic or Latino (Hispanic), and multiracial persons; residents of rural counties; and Janssen primary series recipients. Among 58.8 million eligible first booster dose recipients aged ≥50 years, 20.0 million (34.0%) received a second booster dose. Second booster dose coverage was lowest among persons aged 50–64 years; males; Hispanic, Black, and multiracial persons; residents of rural counties; and Janssen primary series recipients. Interventions focused on improving public health communication and outreach to populations with low booster and second booster dose vaccination coverage should be developed to increase access to COVID-19 vaccines and ensure that all persons can benefit from the increased protection conferred by COVID-19 vaccine booster doses.

On August 13, 2021, CDC’s Advisory Committee on Immunization Practices (ACIP) recommended that moderately or severely immunocompromised persons receive an additional dose to complete a primary series of Moderna (persons aged ≥18 years) or Pfizer-BioNTech (persons aged ≥12 years) COVID-19 vaccine (Supplementary Table, https://stacks.cdc.gov/view/cdc/120701). On September 24, and October 21, 2021, a COVID-19 booster dose was recommended for selected populations aged ≥18 years,* and then recommended for all persons aged ≥18 years on November 19, 2021. On December 9, 2021, January 5, 2022, and May 19, 2022, booster dose recommendations were expanded to Pfizer-BioNTech recipients aged 16–17, 12–15, and 5–11 years, respectively. In addition, selected populations, including all persons aged ≥50 years and moderately or severely immunocompromised persons aged ≥12 years, became eligible to receive a second COVID-19 booster dose on March 29, 2022.

Data on COVID-19 vaccine administration in the United States are reported to CDC by jurisdictions, pharmacies, and federal entities.^†^ COVID-19 vaccine doses administered during August 13, 2021–August 5, 2022, among persons aged ≥5 years in 50 states (excluding persons aged <18 years in Idaho)^§^ and DC, were analyzed to assess booster and second booster dose vaccination coverage by age group, sex, race and ethnicity, urban-rural classification,^¶^ and the primary series vaccine product received. Booster dose vaccination coverage was calculated among persons who completed a primary series** of Moderna, Pfizer-BioNTech, or Janssen COVID-19 vaccine and were eligible to receive a booster dose by the end of the analysis period.^††^ Persons who received 2 mRNA COVID-19 doses or 1 Janssen COVID-19 dose were defined as having completed a primary series because data to identify persons who might have received an additional primary dose were not available. A booster dose was defined as a homologous or heterologous dose of COVID-19 vaccine administered ≥4 weeks^§§^ after completion of a primary series. A second booster dose was defined as a homologous or heterologous dose of COVID-19 vaccine administered ≥3 months (mRNA primary series recipients) or ≥2 months (Janssen recipients) after receipt of the first booster dose.

Information on recipient race and ethnicity was available for 73.6% of the eligible population. Analyses were conducted using SQL Server Management Studio (version 18; Microsoft) and SAS software (version 9.4; SAS Institute). Tests for statistical significance were not conducted because these data are reflective of the U.S. population aged ≥5 years^¶¶^ and were not based on probability population samples. This activity was reviewed by CDC and was conducted consistent with applicable federal law and CDC policy.***

As of August 5, 2022, 214.4 million persons aged ≥5 years (68.6% of the U.S. population aged ≥5 years)^†††^ were eligible to receive a booster dose. Among this population, 106.3 million (49.6%) received a booster dose (Table 1). Booster coverage increased with age, ranging from 15.6% among children aged 5–11 years to 69.5% among adults aged ≥65 years. Booster coverage was lower among males (47.3%) than among females (51.9%), and the coverage difference between males and females was largest among persons aged 18–39 years (6 percentage points). Overall, booster coverage varied by race and ethnicity, ranging from 37.3% among Hispanic persons to 58.5% among non-Hispanic Asian persons. When stratified by age group, the lowest booster dose coverage among persons aged 5–39 years was among Black persons (range = 9.8%–27.9%), and among those aged ≥40 years, coverage was lowest among Hispanic (range = 45.4%–64.0%) and multiracial (range = 45.7%–62.7%) persons. Booster dose coverage was lower in persons living in rural counties (micropolitan and noncore) (48.5%) than among urban residents (50.3%), although coverage differences by urban-rural classification were smaller among older adults. Among persons aged ≥18 years, booster coverage among Janssen, Moderna, and Pfizer-BioNTech primary series recipients was 34.8%, 56.3%, and 51.9%, respectively.

**TABLE 1:** Characteristics of COVID-19 booster dose vaccination recipients aged ≥5 years as a percentage of the eligible population* aged ≥5 years, by age group, sex,^†^ race and ethnicity,^§^^,^^¶^ urban-rural classification,** and primary series vaccine product^††^ — United States, August 13, 2021–August 5, 2022

Characteristic	No. (% of eligible population) vaccinated, by age group, yrs					
	Total	5–11	12–17	18–39	40–64	≥65
**No. of eligible persons**	**214,371,606**	**7,579,057**	**14,373,389**	**63,832,354**	**80,437,874**	**48,148,932**
**Total vaccinated**	**106,252,812 (49.6)**	1,181,821 (15.6)	4,795,396 (33.4)	23,971,719 (37.6)	42,827,799 (53.2)	33,476,077 (69.5)
**Sex**						
Female	**57,838,625 (51.9)**	584,423 (15.6)	2,514,377 (34.6)	13,254,320 (40.5)	23,022,647 (55.4)	18,462,858 (70.3)
Male	**47,968,126 (47.3)**	595,704 (15.6)	2,270,715 (32.1)	10,589,555 (34.6)	19,598,577 (51.3)	14,913,575 (68.8)
**Race and ethnicity**						
AI/AN	**653,776 (45.2)**	10,277 (13.4)	50,881 (34.0)	166,569 (36.0)	273,904 (52.4)	152,145 (65.0)
Asian	**6,790,145 (58.5)**	152,571 (20.6)	489,722 (51.2)	2,312,672 (54.2)	2,657,574 (65.2)	1,177,606 (75.5)
Black or African American	**7,361,157 (42.9)**	55,840 (9.8)	323,974 (24.1)	1,389,409 (27.6)	3,475,085 (49.2)	2,116,849 (67.3)
Hispanic or Latino	**11,530,086 (37.3)**	156,334 (10.4)	892,558 (25.4)	3,446,614 (29.4)	4,966,386 (45.4)	2,068,194 (64.0)
NH/OPI	**226,574 (46.7)**	3,380 (15.7)	14,587 (33.9)	63,345 (36.2)	99,454 (55.7)	45,808 (68.8)
White	**50,903,937 (54.7)**	528,485 (17.7)	2,078,786 (37.8)	9,926,219 (41.5)	19,457,448 (56.8)	18,912,999 (71.8)
Two or more races	**1,269,279 (40.5)**	36,180 (17.9)	109,183 (33.0)	337,384 (31.6)	470,782 (45.7)	315,750 (62.7)
Unknown or other race	**27,517,858 (48.6)**	238,754 (16.2)	835,705 (32.9)	6,329,507 (36.9)	11,427,166 (51.0)	8,686,726 (66.5)
**Urban-rural classification, two-level**						
Urban	**91,471,674 (50.3)**	1,072,472 (16.0)	4,356,807 (34.3)	21,467,699 (38.8)	37,002,405 (54.4)	27,572,291 (70.6)
Rural	**11,237,723 (48.5)**	50,278 (10.2)	308,437 (25.7)	1,630,043 (30.2)	4,351,229 (49.2)	4,897,736 (67.7)
**Urban-rural classification, six-level**						
Large central metro	**34,904,526 (50.4)**	473,646 (17.7)	1,705,095 (34.6)	9,549,773 (40.8)	14,158,318 (55.2)	9,017,694 (71.3)
Large fringe metro	**27,729,051 (51.6)**	344,640 (16.1)	1,467,727 (37.0)	6,023,947 (39.8)	11,580,198 (55.6)	8,312,539 (71.1)
Medium metro	**20,741,562 (49.1)**	194,202 (13.6)	896,992 (31.3)	4,334,684 (35.6)	8,175,484 (52.7)	7,140,200 (69.6)
Small metro	**8,096,535 (49.2)**	59,984 (13.5)	286,993 (30.0)	1,559,295 (34.0)	3,088,405 (51.6)	3,101,858 (69.4)
Micropolitan	**6,802,071 (48.2)**	34,556 (10.6)	205,991 (26.6)	1,072,177 (30.8)	2,654,387 (49.6)	2,834,960 (67.9)
Noncore	**4,435,652 (49.1)**	15,722 (9.6)	102,446 (24.0)	557,866 (29.3)	1,696,842 (48.7)	2,062,776 (67.5)
**Primary series vaccine product**						
Janssen (Johnson & Johnson)	**5,858,549 (34.8)**	NA	NA	1,698,326 (26.2)	3,091,323 (38.6)	1,068,900 (45.7)
Moderna	**42,029,340 (56.3)**	NA	NA	8,546,104 (40.6)	17,190,419 (56.1)	16,292,817 (70.9)
Pfizer-BioNTech	**58,364,923 (47.5)**	1,181,821 (15.6)	4,795,396 (33.4)	13,727,289 (37.8)	22,546,057 (54.0)	16,114,360 (70.6)

**Abbreviations**: AI/AN = American Indian or Alaska Native; NA = not applicable; NH/OPI = Native Hawaiian or other Pacific Islander.

* The eligible population is defined as persons aged ≥5 years who completed a primary COVID-19 vaccination series and were eligible to receive a booster dose by the end of the analysis period. For Pfizer-BioNTech and Moderna primary series recipients, 2 primary series doses must have been received by March 5, 2022 (≥5 months earlier); for Janssen recipients, 1 primary series dose must have been received by June 10, 2022 (≥2 months earlier).

^†^ Information on the recipient's sex was not available for 0.7% (1,476,563) of the population with a completed primary series. Among these, 446,061 persons received a booster dose.

^§^ AI/AN, Asian, Black or African American, NH/OPI, and White persons, and persons of one or more races were non-Hispanic or Latino; Hispanic or Latino persons could be of any race.

^¶^ Information on the recipient's race or ethnicity was not available for 26.4% (56,637,652) of the population with a completed primary series. Among these, 27,517,858 persons received a booster dose.

** Information on the recipient's county of residence was not available for 4.4% (9,459,737) of the population with a completed primary series. Among these, 3,543,415 persons received a first booster dose.

**^††^** For Pfizer-BioNTech primary series recipients, the total booster coverage was calculated among persons aged ≥5 years, whereas the total booster coverage for Moderna and Janssen primary series recipients was calculated among persons aged ≥18 years. The total booster coverage for Pfizer-BioNTech primary series recipients aged ≥18 years is 51.9%.

Among 58.8 million persons aged ≥50 years who were eligible to receive a second booster dose, 20.0 million (34.0%) received a second booster by August 5, 2022 (Table 2). Second booster dose coverage increased with age, ranging from 26.1% among persons aged 50–64 years to 41.4% among those aged ≥75 years. Second booster dose coverage was lowest among males, Hispanic and Black persons, persons living in rural counties, and Janssen primary series recipients.

**TABLE 2:** Characteristics of COVID-19 second booster dose vaccination recipients aged ≥50 years as a percentage of the eligible population aged ≥50 years with a first booster dose,* by age group, sex,^†^ race and ethnicity,^§^^,^^¶^ and urban-rural classification** — United States, January 13, 2022–August 5, 2022

Characteristic	No. (% eligible population), by age group, yrs			
	Total	50–64	65–74	≥75
**No. of eligible persons with a first booster dose**	**58,816,621**	**27,276,149**	**18,943,334**	**12,597,138**
**Total vaccinated**	**19,974,129 (34.0)**	7,108,294 (26.1)	7,650,363 (40.4)	5,215,472 (41.4)
**Sex**				
Female	**11,047,047 (34.6)**	3,902,233 (26.7)	4,171,696 (41.1)	2,973,118 (41.1)
Male	**8,875,045 (33.3)**	3,182,593 (25.4)	3,461,044 (39.6)	2,231,408 (41.9)
**Race and ethnicity**				
AI/AN	**97,664 (31.7)**	45,041 (27.0)	34,100 (36.9)	18,523 (38.2)
Asian	**906,530 (36.1)**	399,363 (28.2)	307,002 (44.5)	200,165 (49.5)
Black or African American	**1,182,553 (28.1)**	494,591 (22.0)	452,565 (34.7)	235,397 (36.0)
Hispanic or Latino	**1,137,781 (24.4)**	541,812 (19.6)	383,202 (31.3)	212,767 (31.5)
NH/OPI	**32,196 (32.6)**	15,085 (27.0)	11,112 (39.6)	5,999 (40.9)
White	**11,359,753 (36.6)**	3,678,835 (28.1)	4,519,386 (42.7)	3,161,532 (43.0)
Two or more races	**191,434 (33.7)**	74,772 (27.0)	70,809 (40.2)	45,853 (39.9)
Unknown or other race	**5,066,218 (32.8)**	1,858,795 (25.6)	1,872,187 (38.7)	1,335,236 (40.0)
**Urban-rural classification, two-level**				
Urban	**17,110,072 (34.7)**	6,228,966 (26.7)	6,500,217 (41.6)	4,380,889 (42.5)
Rural	**2,326,717 (30.1)**	671,880 (22.0)	953,884 (34.8)	700,953 (36.3)
**Urban-rural classification, six-level**				
Large central metro	**6,015,482 (35.5)**	2,385,983 (27.9)	2,187,784 (42.6)	1,441,715 (43.8)
Large fringe metro	**5,311,941 (34.9)**	1,963,815 (26.6)	2,002,077 (42.3)	1,346,049 (43.3)
Medium metro	**4,137,088 (34.3)**	1,376,505 (25.9)	1,641,235 (40.7)	1,119,348 (41.4)
Small metro	**1,645,561 (32.8)**	502,663 (24.3)	669,121 (38.5)	473,777 (39.4)
Micropolitan	**1,393,949 (30.8)**	412,877 (22.5)	571,693 (35.9)	409,379 (37.1)
Noncore	**932,768 (29.3)**	259,003 (21.3)	382,191 (33.4)	291,574 (35.3)
**Primary series vaccine product**				
Janssen (Johnson & Johnson)	**645,707 (20.7)**	410,836 (19.8)	162,852 (23.0)	72,019 (21.1)
Moderna	**9,187,651 (34.6)**	2,903,685 (26.0)	3,730,327 (40.6)	2,553,639 (41.3)
Pfizer-BioNTech	**10,140,771 (34.8)**	3,793,773 (27.1)	3,757,184 (41.5)	2,589,814 (42.7)

**Abbreviations**: AI/AN = American Indian or Alaska Native; NH/OPI = Native Hawaiian or other Pacific Islander.

* The eligible population is defined as persons aged ≥50 years at time of primary series completion who received a first booster dose and were eligible to receive a second booster dose by the end of the analysis period.

^†^ Information on the recipient's sex was not available for 0.4% (223,377) of the population with a first booster dose. Among these, 52,037 persons received a second booster dose.

^§^ AI/AN, Asian, Black or African American, NH/OPI, and White persons, and persons of one or more races were non-Hispanic or Latino; Hispanic or Latino persons could be of any race.

^¶^ Information on the recipient's race or ethnicity was not available for 26.3% (15,446,958) of the population with a first booster dose. Among these, 5,066,218 persons received a second booster dose.

** Information on the recipient's county of residence was not available for 3.2% (1,859,993) of the population with a first booster dose. Among these, 537,340 persons received a second booster dose.

## Discussion

By August 5, 2022, approximately one half of the eligible population aged ≥5 years had received a COVID-19 vaccine booster dose, representing approximately one third (34.0%) of the U.S. population aged ≥5 years. Booster and second booster dose vaccination coverage rates were lowest among the youngest age groups; males; Black, Hispanic, and multiracial persons; residents of rural counties; and Janssen primary series recipients. Some similarities existed between booster dose coverage and primary series coverage trends as of August 21, 2022, with children, adolescents, younger adults aged 18–24 years, males, and Black persons being underrepresented among fully vaccinated persons (*5*).

Booster dose coverage was highest among adults aged ≥65 years (69.5%), with smaller coverage differences across sex, race and ethnicity, and urban-rural classification compared with that in adults aged 18–64 years. Among age groups, the lowest booster dose coverage was among children aged 5–11 years (15.6%), followed by that among adolescents aged 12–17 years (33.4%). Children aged 5–11 years were recommended to receive a booster dose most recently, which might partially explain the low coverage in this group. Racial and ethnic disparities in booster dose coverage were largest (≥26 percentage points) among persons aged 12–39 years. Understanding the factors contributing to low booster and second booster dose coverage among Black, Hispanic, and multiracial populations, and designing interventions to address these factors, is crucial to ensuring equitable access to COVID-19 vaccination.

Booster and second booster dose coverage rates among Janssen primary series recipients were lower than those among mRNA vaccine recipients. One possible reason for this is the Janssen 1-dose primary series might have been preferred by persons less likely to receive multiple doses, such as transient populations (e.g., persons experiencing homelessness), persons with limited access to health care, and persons with needle aversion. Booster and second booster dose coverage was lower among residents of rural counties than that among urban residents; lower COVID-19 vaccine acceptance has been observed in rural areas, and rural residents might also experience more barriers to accessing health care than do urban residents (*6*). Persons living in rural areas were previously found to be less likely to engage in COVID-19 preventive behaviors such as mask wearing (*7*), which would likely increase the potential benefit provided by a booster dose in this population.

The findings in this report are subject to at least five limitations. First, COVID-19 vaccine booster dose recommendations were released during a 10-month period, and some populations had less time than others to receive a booster dose. Further, changes in COVID-19 variant predominance and case prevalence during this period likely affected booster and second booster dose acceptance among different populations. Second, misclassification of vaccination status might have occurred if linkage among vaccination records in jurisdiction-specific data systems was not possible, if, for example, persons received doses in different jurisdictions. Third, eligibility was determined by age at primary series completion, and a small number of persons who met the minimum eligible age requirement after primary series completion might have been excluded. Fourth, a small proportion of booster and second booster doses might have been misclassified because information on immunocompromise status was not available to identify immunocompromised persons who might have received an additional primary series dose. In addition, misclassification might have occurred due to the definitions for booster and second booster doses, which were designed to include doses administered to immunocompromised persons. However, after receipt of a primary series, approximately 99.0% of persons who received 1 subsequent dose received this dose after the minimum recommended interval for a booster dose; 99.6% of persons who received 2 subsequent doses received the second postprimary series dose after the minimum recommended interval for a second booster dose.^§§§^ Finally, race or ethnicity was unknown, unable to be reported, or invalid for approximately one quarter of the population, which could bias results. In May 2022, the National Immunization Survey Adult COVID Module (NIS-ACM) found no substantial racial and ethnic disparities among fully vaccinated adults (*8*); however, disparities across race and ethnicity were present in booster dose coverage based on NIS-ACM.

All fully vaccinated eligible persons aged ≥5 years are recommended to receive a COVID-19 booster vaccine dose, and certain populations, including adults aged ≥50 years, are recommended to receive a second booster dose when eligible (*4*). Booster doses increase the primary series vaccine effectiveness and strengthen the immune response in children, adolescents, and adults (*1*–*3*). Health care providers can educate and encourage all persons to receive a booster dose when they are eligible. Focused interventions should be developed and implemented to improve access to COVID-19 vaccines and ensure the effectiveness of public health communication and outreach to populations with low coverage, which might reduce health disparities.

**Summary**

**What is already known about this topic?**

A COVID-19 vaccine booster dose provides enhanced protection against SARS-CoV-2 infection, COVID-19–associated emergency department visits, hospitalization, and death.

**What is added by this report?**

Among 214 million eligible persons aged ≥5 years, approximately one half received a booster dose. Among 55 million eligible persons aged ≥50 years, approximately one third received a second booster dose. Booster and second booster dose coverage rates were lower among the youngest age groups; males; non-Hispanic Black or African American, Hispanic or Latino, and multiracial persons; residents of rural counties; and Janssen (Johnson & Johnson) primary series recipients.

**What are the implications for public health practice?**

Focused interventions to improve vaccine equity and effectiveness of outreach to populations with low booster and second booster dose coverage should be developed and implemented.

## Footnotes

* The September 24, 2021, booster dose recommendations included selected Pfizer-BioNTech primary series recipients aged ≥18 years. The October 21, 2021, booster dose recommendations included selected Moderna primary series recipients aged ≥18 years and all Janssen primary series recipients aged ≥18 years.

† Data were regularly reported to CDC via immunization information systems (IISs), the Vaccine Administration System, or direct data submission. Timely reporting from COVID-19 vaccine providers to jurisdictional data systems is required. The IIS jurisdictions included in this analysis comprise the 50 U.S. states and six local jurisdictions (Chicago, Illinois; Houston, Texas; San Antonio, Texas; Philadelphia, Pennsylvania; New York, New York; and DC).

§ Aggregate data are submitted for vaccine doses administered in Idaho to persons aged <18 years. These data could not be included in the analysis because linkage between primary series and booster doses was not possible.

¶ The vaccine recipient’s county of residence was classified using the 2013 National Center for Health Statistics Urban-Rural Classification Scheme for Counties. Urban counties include counties assigned to four metropolitan levels (large central metropolitan, large fringe metropolitan, medium metropolitan, and small metropolitan), whereas rural counties are those assigned to two nonmetropolitan levels (micropolitan and noncore). Additional information is online. https://www.cdc.gov/nchs/data_access/urban_rural.htm

** During the analysis period, the Food and Drug Administration–approved or authorized COVID-19 vaccines with a booster dose recommendation were Moderna (persons aged ≥18 years), Pfizer-BioNTech (persons aged ≥5 years), and Janssen (persons aged ≥18 years). To be considered to have completed a primary series, persons must have received 2 primary series doses of mRNA vaccine on different days or received 1 dose of Janssen primary series vaccine; 2-dose mRNA primary series recipients were categorized by the vaccine product received for the second dose of the primary series.

†† Eligibility was determined by age at time of primary series completion and date of primary series completion. To be considered part of the eligible population, persons must have received the second dose of a primary series of mRNA vaccine ≥5 months before the end of the analysis period (by March 5, 2022) or received 1 primary series dose of Janssen vaccine ≥2 months before the end of the analysis period (by June 10, 2022).

§§ A 4-day grace period was subtracted from all interval calculations to allow for doses received ≤4 days earlier than recommended.

¶¶ Excluding persons in Idaho aged <18 years.

*** 45 C.F.R. part 46.102(l)(2); 21 C.F.R. part 56; 42 U.S.C. Sect. 241(d); 5 U.S.C. Sect. 552a; 44 U.S.C. Sect. 3501 et seq.

††† U.S. population estimates for persons aged ≥5 years came from the U.S. Census Bureau’s 2021 Population Estimates Program and excluded persons in Idaho aged <18 years to reflect the population under analysis.

§§§ Calculations of the interval between primary series completion and postprimary series doses were available for 91.8% of booster dose recipients and 94.5% of second booster dose recipients (excluding data received via direct data submission). In these calculations, the minimum recommended interval for a booster dose was defined as 5 months between primary series completion and administration of the first postprimary dose; the minimum recommended interval for a second booster dose was defined as 4 months between administration of the first postprimary series dose and the second postprimary series dose.
